# Optimising editorial expertise

**DOI:** 10.1007/s12471-024-01871-x

**Published:** 2024-04-23

**Authors:** Pim van der Harst

**Affiliations:** Department of Cardiology, Division of Heart & Lungs, University Medical Center Utrecht, Utrecht, The Netherlands

In line with our ambition set out earlier this year [1], I am pleased to introduce significant changes in the listing of the associate editors for the *Netherlands Heart Journal*. As it is important for the journal to renew and improve to adapt to the evolving landscape of the cardiology field, we have restructured our editorial board to better reflect the diversity of the cardiology field.

Our associate editorial team is now organised according to specialisation. This strategic decision allows us to ensure expertise across different cardiology areas of interest to our readership. Although we recognise that editors might have more than one expertise, we have made some arbitrary decisions to ensure the majority of categories are covered by at least one associate editor. Future adjustments will be necessary to optimise the alignment between editors and the categories required. Despite some remaining disbalances in the number of editors among the categories, we are confident that by more selective recruitment this will lead to a more balanced editorial board in the future. This proactive approach is important to ensure that we can consistently deliver high-quality content that addresses the full bandwidth of topics published in the *Netherlands Heart Journal*.

Furthermore, we will renew the dynamics of our editorial board by implementing a rotation system for editors. Periodically rotating individuals in and out of their roles introduces fresh perspectives and allows us to better accommodate developments in the field.

In addition, this new policy will allow us to more actively seek to strengthen our connections with different committees and working groups organised within the Netherlands Society of Cardiology (NVVC). Collaborating closely with these groups will not only enhance the relevance of our journal but also foster a more cohesive and interconnected community.

In conclusion, we believe that the restructured editorial board, coupled with increased collaboration with NVVC groups, will enhance the quality, relevance, and impact of the *Netherlands Heart Journal* for years to come.
